# Does the Narrative About the Use of Evidence in Priority Setting Vary Across Health Programs Within the Health Sector: A Case Study of 6 Programs in a Low-Income National Healthcare System

**DOI:** 10.15171/ijhpm.2019.133

**Published:** 2020-01-21

**Authors:** Lydia Kapiriri

**Affiliations:** Department of Health, Aging, and Society, McMaster University, Hamilton, ON, Canada.

**Keywords:** Priority Setting, Use of Evidence, Health System, Low-Income Countries, Uganda

## Abstract

**Background:** There is a growing body of literature on evidence-informed priority setting. However, the literature on the use of evidence when setting healthcare priorities in low-income countries (LICs), tends to treat the healthcare system (HCS) as a single unit, despite the existence of multiple programs within the HCS, some of which are donor supported.

**Objectives:** (i) To examine how Ugandan health policy-makers define and attribute value to the different types of evidence; (ii) Based on 6 health programs (HIV, maternal, newborn and child health [MNCH], vaccines, emergencies, health systems, and non- communicable diseases [NCDs]) to discuss the policy-makers’ reported access to and use of evidence in priority setting across the 6 health programs in Uganda; and (iii) To identify the challenges related to the access to and use of evidence.

**Methods:** This was a qualitative study based on in-depth key informant interviews with 60 national level (working in 6 different health programs) and 27 sub-national (district) level policy-makers. Data were analysed used a modified thematic approach.

**Results:** While all respondents recognized and endeavored to use evidence when setting healthcare priorities across the 6 programs and in the districts; more national level respondents tended to value quantitative evidence, while more district level respondents tended to value qualitative evidence from the community. Challenges to the use of evidence included access, quality, and competing values. Respondents from highly politicized and donor supported programs such as vaccines, HIV and maternal neonatal and child health were more likely to report that they had access to, and consistently used evidence in priority setting.

**Conclusion:** This study highlighted differences in the perceptions, access to, and use of evidence in priority setting in the different programs within a single HCS. The strong infrastructure in place to support for the access to and use of evidence in the politicized and donor supported programs should be leveraged to support the availability and use of evidence in the relatively under-resourced programs. Further research could explore the impact of unequal availability of evidence on priority setting between health programs within the HCS.

## Background

Evidence^[1]^, in its myriad forms, is perceived as a critical part of policy-making in health.^1,2^ Priority setting is a critical process within the health policy-making and implementation cycle. Systematic healthcare priority setting^[2]^ involves an explicit process of selecting which health interventions to pursue, for the purposes of resource allocation. According to the literature, using evidence (eg, burden of disease, cost-effectiveness, and cost-benefit assessments) when setting healthcare priorities promotes objectivity, improves consistency, and strengthens the validity of the prioritization process and the decisions. The result is an improved quality of decisions.^3,4^ The use of evidence in priority setting is therefore critical, especially in low-income countries (LICs) where resources are scarce.^5-8^

There have been several global level calls to improve evidence-informed policy-making. For example, the statement from the 2004 Ministerial Summit on Health Research in Mexico City; the resolution from the 58th World Health Assembly which urged member states to strengthen knowledge transfer mechanisms; the 2008 Bamako Statement that called for countries to ensure that decision-makers have access to relevant evidence^1,2^; the World Report on Knowledge for Better Health^9^ and the latest call which came with the sustainable development goals where the collection and dissemination of high quality evidence is recognized as a key element in facilitating effective planning, follow-up and review of the implementation of the 2030 agenda for sustainable development.^10^

The response to these calls has been the development of several initiatives at the global, regional and national that support the generation and use of evidence in decision-making. Examples of global level initiatives include the WHO’s–CHOosing Interventions that are Cost-Effective,^11^ Grading of Recommendations Assessment, Development and Evaluation,^12^ the Disease control priorities project.^13^ At the regional level initiatives such as the Evidence Informed Policy Network,^3^ and the International Decision Support Initiative,^14^ are working to strengthen decision-makers access to and use of evidence in several countries.^3,10^ Efforts at the national level have focused on improving the quality of the available evidence eg, in Ethiopia^15^; as well as facilitating the use of evidence in policy dialogues by conducting rapid reviews and providing readily accessible evidence to policy-makers eg, in Uganda.^10^ There have been concurrent efforts to facilitate evidence based priority setting at the sub-national levels through projects such as the Community and District Empowerment for Scale-up in Uganda^16,17^ and the data-informed platform for health in India, Nigeria and Ethiopia.^18^ To some extent, these strategies have increased the availability and use of evidence, yet there are still circumstances in which evidence is either lacking, inaccessible, underutilized, or lacks credibility.^19^ For example, the limited resources that hamper priority setting in LICs may also contribute to a lack of credible evidence.^19-22^ Furthermore, there are reported cases where evidence has been disregarded by decision-makers whose priorities lie elsewhere.^1,22-24^

Evidence is thought to be key to improving the quality of priority setting and resource allocation decisions.^25^ Since it is often considered to be objective, evidence is thought to provide means through which personal interests and values which may have undue influence on priority setting, can be mitigated.^4,24-28^ However, there is a paucity of literature on how low-income country decision-makers define, access and use evidence when identifying healthcare priorities. To date most of the literature on the use of evidence in LICs has focused on policy-making^1-3,9-11^ and not specifically on priority setting – which is a critical component of the health policy-making cycle. Some of these studies have, for example identified barriers and facilitators of evidence-informed decision-making,^5,14,22,26^ while others have focused on approaches and tools to facilitate the use of evidence^10,12-16^ in LICs. The benefits of using evidence in healthcare priority setting may be particularly critical in LICs where priority setting is thought to be fraught with uncertainties, and can be easily influenced by the interests and agendas of various stakeholders.^24-28^

The meagre literature on the use of evidence in priority setting in LICs has focused on the health sector as a unit. However, in countries such as Uganda, the health sector comprises of different health programs (see Supplementary file 1) which may, in some cases, have additional funding (and priority setting) mechanisms.^29-34^ In many cases, programs with different funding mechanisms have different information gathering and reporting mechanisms, depending on the funding agencies.^34^ Might there be differences in the way different policy-makers conceptualize, value, access and use evidence in their prioritization processes? This paper responds to this question.

The objectives of this paper are (*i*) To examine how Ugandan health policy-makers define and attribute value to the different types of evidence; (*ii*) Based on 6 health programs (HIV, maternal, newborn and child health [MNCH], Vaccines, emergencies, health systems, and non-communicable diseases [NCDs]) to discuss the policy-makers’ reported access to and use of evidence in priority setting across the 6 health programs in Uganda; and (*iii*) To identify the challenges related to the access to and use of evidence. This paper also highlights key points of similarity and difference between the health programs.

## Methods

### Setting

This paper is part of a large study whose purpose was to describe and evaluate priority setting across 6 programs and in 3 districts in Uganda. The large study employed qualitative methods namely key informant interviews and a review of documents. The study was conducted between March 2014 and March 2016 at the national and sub-national levels (districts) in Uganda.

### Study Sample and Sampling Strategy

We sampled respondents who were knowledgeable of priority setting at the national and district levels (policy-makers and development partners-donors). A mixture of purposeful and snowball sampling strategies was used.^30^ At the national level we targeted respondents who were involved in priority setting with focus on specific programs (HIV, MNCH, Vaccines, emergencies, health systems, and NCDs). The initial respondents (policy-makers heading any of the 6 programs), identified subsequent respondents who they thought were knowledgeable. The interviewees were asked to identify additional respondents until we achieved theoretical saturation—no new themes emerged from subsequent interviews.

Three districts were included in the study. The districts were sampled for regional representation and duration since the district was formed (the old [more than 20 years], the intermediate [over 10 years], and new districts [less than 10 years]). Within each district, we purposed to recruit all members of the district health. Using purposeful and snowball sampling, the initial respondent was the Director of district health services who identified subsequent respondents.

We interviewed a total of 60 respondents at the national level (including development assistance partners (DAPs) or donors and policy-makers) and 27 at the sub-national (district) level (Table).

**Table:** Respondents by Program and District

**Program**	**Number of Respondents**
**National Level Respondents**	
HIV	16
MNCH	9
Vaccines	7
NCDs	5
Emergencies	8
Health systems	15^a^
Sub-total	60
**Districts Level Respondents**	
District A	5
District B	7
District C	15^b^
Sub-total	27

Abbreviations: MNCH, maternal, newborn and child health; NCD, non-communicable disease. ^a^Health systems respondents also spoke to almost all the other cases; but were specifically asked about the health system. ^b^District C was the oldest district, it had all their administrative positions filled as compared to the newer districts.

The reviewed documents included 3 health sector strategic plans (2000-2015), 14 Annual health sector reports (2001-2015), 2 National health policy statements (1999-2010), Meeting minutes (2009-2015) and media reports (2005-2015). These were aligned with the health strategic planning cycles.

## Data Collection and Analysis

The interviews were conducted by a trained research assistant and the principle investigator using a pilot-tested interview guide (see Supplementary file 2). The interview guide included: a definition of priority setting and its relevance, followed with general questions about the priority setting process and specific questions and probing about the use of evidence in the prioritization process. Interviews were conducted in English language and lasted between 30-45 minutes. All interviews were audio recorded with permission from the respondents, and were transcribed verbatim.

NVIVO-10 was used to analyze the transcribed data. Three members of the research team coded one interview; after the independent coding, they met and discussed the code names each identified from the interview. Codes that were consistent were adopted for use; codes where there was no agreement were discussed and an agreed upon code identified. The edited list of codes was then used for to guide the coding the rest of the interviews.^35,36^ However, an open stance was maintained throughout the process and any codes that emerged during the process were continually discussed and agreed on codes were added to the code book. While several themes about priority setting emerged from the data (see Supplementary file 3), this paper focuses on the “evidence” theme. In the analyzing this theme, the following dimensions of evidence emerged from the data: *definition of evidence; Value of different types of evidence; sources of evidence; role/use of evidence; related challenges.* Further analysis involved assessing the degree of agreement and divergence between responses from the different programs and the levels of decision-making. Initial interpretations of the findings were presented, discussed and validated by the policy-makers in 2017.

## Results

The first section is organized according to the categories that emerged from the data namely; “definitions of evidence,” “the value of evidence,” “the sources of evidence,” “the role of evidence in priority setting” and “the challenges with the access to and use of evidence. The last section of the results provides an analysis of the relationship between the different categories and sub-categories. Illustrative quotes are provided in text and in an additional file (see Supplementary file 4). Identifiers for the quotes include the designation of the respondent vis: Respondents from the Ministry of health: MoH, the DAPs/donors: DAP, the district respondents: D; this is followed by a randomly allocated number.

### Definitions of Evidence

Respondents were not provided with a definition for evidence, hence one theme that emerged from analyzing the theme on evidence was the variety of terms that interviewees involved in this study used to refer to evidence. As explained by one respondent;

“*what constitutes as ‘evidence’ for one person may not be viewed as appropriate evidence by another*…” (DAP_1).

As such, terminology used by the respondents ranged from: objective terms such as *data* and *statistics* on one hand, and subjective terms such as *personal knowledge* and *experience* on the other. For some respondents, evidence encompassed everything from quantifiable epidemiological data to subjective immeasurable concepts such as well-being. In their responses, respondents focused on the aspects of evidence that they value most. Broadly speaking, however, their uses of the term can be categorized as quantitative or qualitative, with national-level respondents prioritizing the former and sub-national–level respondents tending to prefer the latter. Both types of evidence are discussed below.

#### Quantitative Evidence

Overall, respondents showed a preference for evidence with easily collectable and analyzable metrics. These also reported that they privileged quantitative evidence because it was easily understood. As they explained, quantitative information such as economic evidence, mortality and morbidity rates, and burden of disease are (*a*) comparatively easy to measure (*b*) favorable to politicians and (*c*) well correlated with opportunities for future funding:

*“…You have to show them (the politicians) figures. When they see the numbers you get on the list (of priorities)...”* (D3_2).

*“…You have to measure the benefits and the cost of any program and if the benefits are greater than the cost then it’s a priority, you should do it...”* (DAP_ HIV_2).

At the national level, some respondents reported a reliance on broadly generalizable data, which can be applied across countries and regions.

*“…So most people are not using evidence, what most people are relying on is what the global conventions said we should do…”* (MOH_Vaccines_1).

However, some respondents noted that contextualizing the quantitative data provided by the international community can be difficult without the necessary technical competence, which further contributes to their reliance on international experts in making use of data.

#### Qualitative Evidence

Unlike national-level respondents, who tend to focus on one program, sub-national decision-makers tend to focus on evidence that can help facilitate program implementation across a range of areas within the healthcare system (HCS). Three district level respondents in this study noted that it is important to look beyond the quantitative evidence if priority setting and program implementation are to succeed. These gave an example of HIV whereby they alleged that successful interventions must incorporate cultural preferences and political demands alongside measurable factors such as effectiveness. If costs were the sole metric by which priorities were set, abstinence-based education would be the best approach for HIV prevention. However, a more complex picture, that requires decision-makers to account for a range of political and socio-cultural needs, emerges when information such as beneficiary assessment data and cultural preferences are considered.

Acknowledging this, 4 district level respondents mentioned the value of data that incorporates local views, which can be collected through approaches such as conversations between the public and service providers; through the involvement of community resource persons; via suggestion boxes at health facilities, exit interviews in clinics, and community satisfaction surveys.

However, some respondents were concerned about the difficulties in assessing the quality of and integrating this qualitative information in actual decision-making;

*“….I know that there have been big discussions in the organization related to how to try to measure a contribution to for example all activities on communication for development that are related to changing behavior, other things which are also very difficult to measure beyond process indicators* …*”* (DAP_HIV_3).

#### The Sources of Evidence

Respondents identified several sources of the evidence they used in priority setting at the national and district levels. These included; Policy documents and Reports; Special research and “experts.”

#### Policy Documents and Reports

Respondents identified several policy documents and reports they used as sources of evidence for healthcare prioritization. These included national health policy and planning documents, Strategic plans, annual review reports and national statistics. Respondents also identified specific performance reports generated through monitoring and evaluation mechanisms such as the Health management information systems from the districts and lower levels, biannual reviews (one respondent), in addition to monitoring indicators set in strategic and work plans (one respondent). Furthermore, respondent discussed large-scale sector reviews eg, the Health Financing Review, (which explored 11 domains including health financing, performance, and efficiency of interventions) and the National Health Accounts survey done in 2009/2010 which provided trends and relative proportions of government outlays, donor outlays but also other sources of funding like Out of pocket expenditure.

“…*the performance reports are available – the Ministry does performance evaluation every 6 months – we used to do it quarterly but found that it was a bit too frequent”* (MOH_HIV_3).

#### Research

Respondents discussed research and special studies as an additional source. Specifically, several respondents discussed how the results from the burden of disease study though dated, still played an important role. Other specific studies include beneficiary assessments and specific program reviews and assessments;

*“The global burden of disease study, the burden of disease study that was done for Uganda in 1993 still influences very much the priorities and the interventions therein that are used for the health sector…”* (DAP_HS_2).

*“Well it is the middle level and high level managers within the organization who regularly review program data and based on that program data they’re going to identify priorities...”* (DAP_MNCH_5).

Furthermore, interviewees articulated using locally produced evidence in the form of statistical assessments of the resources, databases, and other studies conducted at the different levels within the health sector, pilot studies (for example the malaria interventional studies and performance based financing studies), consumer satisfaction and governance surveys. In addition to these, respondents reported using evidence on the successes and failures experienced by similar African countries for transposable interventions, programs, and lessons.

*“…We can also look at the East African community in general how are the other member countries performing? Like in Rwanda, how have they improved their health indicators? We see if we can cut out (implement) those kinds of interventions, maybe we can (also) improve”* (MOH_ HS_12).

In contrast some respondents discussed the significance of international evidence;

*“… International evidence plays a major role in driving local action and…maybe for many reasons (such as) it is published in the lancet, the WHO has endorsed it…”* (MOH_NCD_3).

While they recognized the importance of international evidence, respondents also highlighted the need for increased support for systematic gathering and use of local evidence. This limitation was emphasized by the district level respondents who talked about how limited resources hampered the generation of local evidence.

#### Experts

Consultative meetings with local and international experts were undoubtedly the most commonly stated medium through which evidence, expertise, and recommendations were generated. These meetings occur between diverse and numerous stakeholders within the Uganda health sector, including but not limited to: donors and other development partners, experts and specialists, district-level health officials, service- and care-providers, and community members/service users. The purpose of these meetings are to generate thorough knowledge of the health sector performance, understand where action is required, and to determine the most appropriate interventions;

*“…technical working groups…people come with presentations, people come with a new areas of research, people come with new evidence…most of the work on priority setting is done at the technical working group…by experts, knowledgeable people that’s where most work is done”* (MOH_ MNCH_9).

*“At community level we create a forum for communities to have an honest discussion with the service providers and make sure that what they are doing responds to their needs”* (DAP_HIV_4).

DAPs, based on their technical and extensive infrastructure, were somehow expected to provide and to facilitate the gathering of evidence:

*“…Evidence is a big issue and it should be, from a technical perspective, it’s really their (DAPs) role to … strengthen and build that evidence base in order to provide it during the resource allocation, prioritization processes…”* (MOH_HIV_1).

The above sources of evidence were corroborated by respondents from the districts, who in addition to the above sources, and possibly due to their proximity to the community, identified the public (through their local representatives), and non- government organizations as additional sources of evidence.

#### The Role of Evidence in Priority Setting

When discussing the role of evidence when setting priorities, respondents from the national and district levels had varied perspectives. While several reported specific aspects of the prioritization process where evidence was reportedly used, some respondents doubted that evidence played a big role in the process.

When discussing how evidence is used in the prioritization processes, the responses were similar at both levels and included;

(*i*) When assessing the effectiveness of program implementation - where monitoring and evaluation evidence is collected,

*“…We majorly use data because data shows the gaps. Remember, yes the resources are limited but our gaps are saying - our data is saying there is a gap, this sub county does not have a maternity ward, we as government go in to make sure the sub county gets a maternity ward…”* (D2_09).

Certain epidemiological indicators can also give insight into how well programs are performing at the local level and can influence priorities that are set by an organization that is involved in implementation. One respondent explicitly described that priorities should be based on the needs of the population, which can be inferred from statistics that reveal gaps in care and service.

“*But at the end of the day, as I said, we need to know how quickly do you diagnose a patient, how much time do you ensure you put in to getting them treated and followed up to completion. So the indicator in that case would be case detection rate. Case identification rate, extreme access rate, cure rate, and the others...”* (DAP_HIV_5).

(*ii*) Evidence was also reported to be used; to guide strategic planning, when identifying priority interventions, and in advocating for more resources.

The use of evidence in planning was reiterated by district level respondents who provided additional examples of how they use evidence to identify and prioritize underperforming facilities,

*“The biostatistician compares up to date records of the entire department per sub-county, per health unit, and in such…we have all the success registered, the failures and the challenges also registered, and then we use such records to guide the planning, to guide priority setting, per facility...*” (D2_2).

(*iii*) Respondents also discussed how evidence was used to improve the quality of their decisions by limiting the role of self- interest. For example, district level respondents discussed how they use evidence to differentiate between the community’s real needs from their expressed needs;

*“…People can express this need but if you go down you’ll find the real need is actually not the one they have expressed…the direction is to always generate evidence to support those expressed needs so that we are able to allocate resources a lot better…”* (D1_1).

Two respondents emphasized the importance of evidence in successful priority setting pointing out the ways in which a lack of evidence can hamper evidence-informed priority setting. Without evidence to set priorities, and without evidence to evaluate the degree to which the priorities are implemented, effective priority setting can be difficult.

#### Challenges Related to the Use of Evidence in Priority Setting

Respondents reported a range of challenges that hinder the use of evidence in priority setting. In particular, they highlighted challenges related to (*i*) the availability and access to evidence, and (*ii*) challenges related to the use of evidence in priority setting, all of which are discussed.

#### Challenges Related to the Availability and Accessibility of Evidence

In discussing the use of evidence for priority setting, lack of evidence was identified as one of the main challenges;

*“….We sometimes set priorities not based on evidence but based on experience. The challenge is to get the data, analyze it and help us in priority setting…”* (MOH_MNCH_5).

Respondents discussed how poor physical infrastructure, political upheaval, and natural disasters can make it difficult for them to reach the areas where data is to be collected. Deficiencies in infrastructure can be particularly problematic when location-specific information is required yet some locations may be inaccessible due to poor physical infrastructure.

As respondent from the emergency program explained,

*“…I find that sometime it is hard for the committees to visit to the community due to lack of the transport…”* (MOH_Emergency_05).

Respondents also discussed financial and personnel related challenges. For example, one national-level respondent highlighted how limited resources can hinder the implementation of country-specific studies. This was discussed in reference to other programs that have more resources:

*“…They (other people) have the resources and people to invest in to do a good job in packaging, delivering, and influencing. If you look at me (from the MOH) what resources do I have to do a good study and disseminate it? Where do I get those resources?”* (MOH_MNCH_1).

While adequate funding has facilitated the availability of evidence for programs such as HIV, inadequate funding, makes it difficult for policy-makers to generate the evidence needed to support decision-making in relatively under resourced programs.

#### Challenges Related to the Actual Use of Evidence in Priority Setting

Respondents discussed several factors that affect the use of evidence. These were either related to the *evidence* (completeness and credibility) or to *the different stakeholders* (interests and the pressure to implement).

#### The Completeness and Credibility of the Evidence

One challenge identified by most of the respondents was the *incompleteness* of the available information. For example, respondents spoke about how they lacked national and sub-national denominators, which numbers would allow priority setters to make inferences about a population:

*“…What we usually have [are] numerators … and you don’t have information for decision-making because you don’t even have the denominators you just have some numbers…”* (MOH_HS_14).

It can be difficult to set priorities that are in the interest of the whole population if basic demographic information is not available to provide a clear picture of the population being served, or if such evidence has been manufactured or distorted.

While some of the respondents recognized the relevance of qualitative evidence in providing a complete understanding of the health problems, they noted that it can be difficult to incorporate evidence that is not easily quantifiable into an evidence-informed priority setting process. However, omitting qualitative evidence means that sometimes decisions are based on “incomplete” quantitative evidence which may not provide a complete picture of the situation.

Although sometimes the decision-makers have access to complete information, the *credibility* of some of the evidence is doubtful. For example, one national-level respondent, talking generally about GAVI funding, reported an instance where they think imaginary numbers were used for funding purposes:

*“… So, it’s happened all the time, if you take GAVI application from countries there are many applications where you have the data on vaccine coverage that were just made up just for the sake of getting money…”* (DAP_Vaccines_6).

Another aspect of the credibility of evidence, is timeliness. Respondents were concerned about having to use out dated information to guide priority setting. These specifically discussed instances where outdated evidence (such as the BOD/CEA study), continues to be used in priority setting.

*“…The global burden of disease study, the burden of disease study that was done for Uganda in 1993 still influences very much the priorities and the interventions therein that are used for the health sector…”* (DAP_ HS_2).

Respondents from the HIV and vaccines programs also cited problems with keeping up with incoming data. In particular, they noted the challenges in staying up-to-date with and subsequently applying the findings from ongoing scientific advances, which evolve at a faster rate than they can process. Conversely, other respondents discussed how sometimes researchers take a long time to generate the required evidence. In such instances, the research evidence tends to be out of synch with decision-makers’ timeframe, hence forcing the policy-makers to make decisions that may not be informed by the most recent evidence:

#### “…A huge challenge is that the implementation and policy-making move at a different pace than research …. I think that there’s always a tension to find better, quicker and more useful ways to [generate research and information] on a continuous cycle because it is … unsatisfactory to only be able to rely on a baseline and an end line, and maybe if you’re lucky a midline, because, over a five-year project, things are moving and changing more rapidly…” (DAP_MNCH_3).

#### Challenges Related to the Different Stakeholders

This section focuses on respondents’ discussions of how donor priorities, government priorities, and local priorities and interests can interfere with the use of evidence in priority setting. There was a feeling among the respondents that donor agendas can lead to a rearrangement of the priorities, even when priorities are based on sound evidence. Governments can also be guilty of using their authority to steer priority setting. Two respondents criticized politicians at both the national and sub-national levels for prioritizing their own interests:

*“…So sometimes the evidence is overlooked …. Budgeting and resource allocation are done from a perspective that looks at balance of delivery—vis-à-vis the perspective of other colleagues on the political scene…”* (D1_3).

To compound this problem, respondents pointed out that well-intentioned politicians who want to make use of evidence for the prioritization process may not always have the capacity to interpret and apply the information at their disposal.

Personal-interests (at the individual or organization level) can also hinder evidence-informed priority setting. National and district level respondents discussed how local partners- especially donors- sometimes push priorities based on their own beliefs, preferences, and needs, in spite of the evidence. Furthermore, they noted that individuals also tend to prioritize health issues that, in one way or the other, has or might affect them.

*“…I think people set priorities when they are endangered. For example, if you feel you are at risk for something then you tend to … put priority on that something …. Ideally, it should be according to the figures, the burden of disease and statistics...”* (MOH_HS_5).

Challenges can also occur in the transition from acquiring evidence to implementing solutions. In particular, respondents highlighted how a desire for quick solutions, and hence implementation, can override the use of evidence. When decision-makers think they know the solutions (possibly based on their experience), generating evidence seems a waste of time and resources. The tendency is to quickly implement the known solutions, even though they are not supported with credible evidence:

*“…People think why waste money doing a lot of appraisals, a lot of studies, a lot of analytical work. We want money for actions because we know what the problems are. But you know what the problems are in a small way, and you do not know how that same problem translates three, four, five years down the road, which is critical, otherwise after four years you’re coming back to the same problem. So, getting that across has been quite a challenge…”* (DAP_HS_5).

A secondary challenge related to the generation and use of evidence in healthcare prioritization is the failure to implement the identified priorities. As implied in the above discussion, policy-makers tend to weigh the costs and benefits of investing in gathering evidence; especially if the possibility that the evidence-informed priorities are not feasible. Logistics eg, limited human resources, can stand in the way of the resultant priorities’ deployment:

*“…For example, we may have … evidence that male circumcision works, and so you want to support the delivery of that service … but you find there are no surgeons. If there is a surgeon, there are no theatre materials. If there are theatre materials, there’s no post recovery management or supplies. You find that the entire system has gaps…”* (DAP_HIV_2).

Just as a lack of resources poses a problem at the start of the evidence-gathering process, a lack of resources or personnel can stand in the way of implementing the solution based on the priorities that have been established.

Despite the obstacles to evidence-informed priority setting, respondents from both the national and sub-national levels reported successes that stemmed directly from using evidence. For example, some discussed how evidence helped priority setters overcome limitations, and how in some projects evidence had been used to successfully establish health priorities. Evidence-weighing processes resulted in the establishment of reproductive health related priorities. Furthermore, the use of evidence related to disease epidemiology, resources, local populations, and cultural practices resulted in successful evidence-informed health system prioritization:

*“…we did a lot of research … to come up with evidence-informed interventions. Starting from baseline in the countries where we were working, we saw, for example, malaria, pneumonia and diarrhea which were the main causes of child mortality, and the most important thing was to really have health workers who were not only specialized doctors but could train in a short period of time to diagnose these illnesses and treat them in an easy way. We found that also culturally, traditional attendants and midwives were very useful for these [purposes], so we said let’s study how effective it would be to prevent illnesses, cure diseases, and save lives by training these kind of health workers…”* (DAP_MNCH_3).

While the results are presented individually and sequentially, they are interrelated. For example, the lack of resources will impact the availability and quality of evidence, its completeness, quality and even the timeliness. Furthermore, how decision-makers define and attribute value to the different kinds of evidence may influence their willingness to invest in gathering either the qualitative or quantitative evidence. The lack of resources may introduce stakeholders with resources, who, by virtue of controlling the resources, also introduce self-interest in setting priorities at the expense of what the evidence dictates. The pressure to implement hampers evidence-informed decision-making. However, the pressure to implement could also contribute to the incompleteness of the evidence – whereby the producers of evidence are also under pressure to produce some kind of evidence for the decision-makers.

## Discussion

We presented findings from an empirical study on how decision-makers at the national and sub-national levels conceptualize, access and perceive the utility of evidence when setting healthcare priorities. Respondents used different terminologies to define evidence. They access the evidence through documents, research and experts. Although there was interest in using evidence, respondents at the national and district levels reported varying challenges related to their access to and use of evidence when setting healthcare priorities.

The study findings that decision-makers have an interest in- and endeavor to use evidence when setting healthcare priorities, in healthcare programming, implementation, and in program evaluation are consistent with the literature on evidence-informed decision-making.^5,8,20,21^ It was beyond the scope of the paper to establish if and how the decision-makers actually used evidence when setting healthcare priorities. Some of the literature on how decision-makers actually use evidence discusses the impact of the decision-making contexts and cases where evidence is used to support already made decisions as opposed to guiding the decision-making processes.^37-40^ However, if the decision-makers we interviewed are genuinely interested in using evidence to guide their prioritization process, the interest may present an opportunity for augmenting the use of evidence, if the identified barriers are addressed. The barrier of limited capacity (human and financial resources) to collect evidence which results in generating of poor quality and incomplete information which in turn affects the use of evidence; have been discussed in the evidence-informed policy-making literature.^41-43^ The importance of good quality and timely evidence cannot be overemphasized. Poor quality of the evidence, timeliness of the evidence, the relevance of the evidence – with emphasis on the context where the decisions are actually made are some of the key limitations to the use of evidence in policy-making in many LICs.^10,14,19,43-47^ While lack of resources may account for the poor quality of evidence, exploring ways through which well-funded programs such as Vaccines—could be leveraged to strengthen the evidence base across the health sector would be beneficial.^48,49^ This is more so since program implementation at the district level is often under the same umbrella and vertical information gathering may prove to be resource intensive.

Since all sub-national (districts) have a health information officer who is responsible for synthesizing district level information, it may be useful that these individuals are supported to effectively accomplish their duties. Capacity strengthening and supporting these individuals to assume leadership in all information gathering within the district, may contribute to more efficient use of the available resources to collect high quality evidence.^47^ At the national level, the existing resource center should be supported with the necessary human resources with the capacity to manage an integrated health information system for all programs.^28,29^ Focused and integrated capacity strengthening and support would alleviate some of the additional barriers of poor quality, completeness, and timeliness.

However, there were subtle differences between responses from the national and sub-national levels and between respondents from the different programs, which are worth discussing.

First, relative to the district level respondents, national level respondents were more likely to conceptualize evidence in terms of quantitative measures and to value this kind of evidence relative to qualitative evidence. The reasons and uses of this kind of evidence (for example as an advocacy tool) may be more critical at the national level.^46^ Most of the resource mobilization and allocation for the health sector occurs at the national level. At that level, measurable, and easily quantifiable evidence tends to be valued, it may be more difficult to make a case based on unquantifiable, let alone measurable problems.^4,45,46^ Furthermore, decision-makers at the national level tend to be appointed technocrats who are relatively removed from the populations they serve.^29,33,50^ Conversely, while some of the sub- national decision-makers are also technocrats, several are elected. Even the technocrats have to be vetted by the political appointees.^50^ There might be pressure for them to listen to, and consider the public’s interests—which tends to be more qualitative. Conversely the sub-national decision-makers are the legitimate implementers of the health programs.^16,29,47,50^ Their proximity to the population and their mandate may compel them to appreciate the qualitative evidence so as to respond to the needs of the populations they serve.

Second, national-level respondents focused on evidence-informed priority setting with respect to policy formulation and program evaluation. By contrast, sub-national–level respondents focused on the use of evidence for the implementation of policy.^16,17^ This means that priority setters at the 2 levels are often using evidence to different ends. Where national-level priority setters rely on evidence to guide higher order decisions about what policies to fund, sub-national–level priority setters are using evidence to determine practical solutions for enacting these national-level policies. As such, there is a gulf separating the uses of evidence even if the evidence being used is the same. This can create multiple demands on a single set of evidence, and can even lead to conflicting ideas about what should be done depending on the ends to which the evidence is interpreted.

Third, there was a perception that the evidence at the sub-national level was of relatively poor quality while that at the national level—especially that collected through reports—was of relatively better quality. This perception was surprising given the ideal hierarchy of decision-making within the decentralization framework. Ideally, the sub-national evidence should be pulled together to form basis for the national level decision-making processes.^29,33,48^ Hence if the sub-national level evidence is of relatively poor quality, it should impact the quality and completeness of the national level evidence.^18,49^ Conversely, this may be a reflection of the differences in the quality of data, between the districts; and the sampling strategy used in the study. The country has over 100 districts and only 3 were included in the study. It is possible that the findings are a reflection on only a small sample which may not impact the overall quality of evidence at the national level—where data from all districts are aggregated. However, this may also be a true reflection of the shortcomings in resources, infrastructure, and personnel capacity at the sub- national level—which need to be addressed.^16,17,43^

The factors that compete and sometimes trump the use of evidence when setting healthcare priorities have been documented in the literature. Some of the literature alludes to the perceptions that factors such as politics, popularity; may, in some cases, be perceived as more critical – at least to the decision-makers – than what the evidence says.^51,52^ In contexts where resources are limited, it is unreasonable to allocate resources according to personalist or organizations’ interests as opposed to the evidence. However, it is also important to note that evidence is just one of the considerations in priority setting. There are additional relevant considerations which may come into play. The literature on the criteria for setting priorities emphasizes the need for enlisting and using criteria that are relevant and acceptable to the priority setting context.^4,53^ It may be problematic if the priorities based on the agreed upon criteria are contrary to what the evidence points to. In some of these cases, unacceptable criteria such as personal interests, come into play. Transparency of the decision-making processes, including the evidence used, the type, and its sources (whose evidence? who was talked to?) and how it is used in the prioritization process would enable decision-makers and all the other stakeholders to evaluate the evidence; against the decisions.^53,54^ Transparency may also deter those who may want to introduce self-interests in the prioritization process.

The findings that there were differences in responses between the different programs that were sampled was interesting. For example, respondents from the HIV, Vaccines and MNCH cases; compared to respondents from for example the NCDs case; were more likely to report the availability of evidence for their prioritization. This finding may not be surprising since these programs have enjoyed external funding which requires stringent accountabilities. In many cases the accountability should be supported with evidence and the funding agencies support the gathering of this evidence.^16,17,30-32,55-58^ As discussed above, it would be efficient for the health system to leverage the resources and the health information structure set up for the funded programs to benefit the less well-funded. Furthermore, respondents from programs such as HIV and Vaccines- seemed more appreciative for qualitative information—which might be a reflection of their experiences with the various types of evidence.^59-61^ Qualitative evidence, which accesses personal experiences play a big role in the acceptability of interventions as were the case of HIV control, and new vaccines.

## Limitations

The findings of this study should be interpreted with caution. Since this was a qualitative study involving a sample of respondents from 6 programs at the national level, and 3 districts, we cannot claim that the findings are generalizable. However, the purpose of the study was to provide in-depth understanding of the use of evidence and not generalization. We however, observed the validity standards for qualitative data collection and reporting and hence believe the findings are credible and make meaningful contribution to the literature on the use of evidence within these programs’ priority setting.

## Conclusion

While there is interest in using evidence in priority setting presents an opportunity for augmenting evidence-informed priority setting, problems with collecting, accessing and interpreting data, and several barriers that make it difficult to translate evidence into meaningful, practical priorities. Hence, decision-makers in LICs need to invest in improving the availability of high quality evidence and committing to its use when setting healthcare priorities. At the district level, there is a need to invest in *systematic* collection of both qualitative and quantitative information right from the community, to the health units. Encouraging the use of this evidence at the level at which it is collected may increase the interest in collecting it. District level information should inform national level priority setting. Similarly, national level decision-makers should strengthen the collection and use of both *qualitative* and quantitative evidence gathered from all the districts within the country. In the mean time, decision-makers should be encouraged to use the based available evidence to improve the quality of the priority setting decisions.

Since lack of quality evidence is more prevalent in some of the health programs (due to different funding models), decision-makers should leverage the resources available in the well-funded programs to harmonize the generation and use of evidence in priority setting across the HCS.

The subtle differences in the availability and use of evidence in priority setting across the health programs within the HCS highlight the need for similar studies in other LIC HCSs to substantiate and understand the nature of these differences and how they may impact actual health system priorities in this era of evidence-informed decision-making and priority setting.

## Acknowledgements

This research was funded by the Canadian Institutes for Health Research, Ottawa, ON, Canada [Grant 10558616]. The author acknowledges the following people for their involvement in various aspects of the project: the study respondents without whose participation, the study would not have been possible, Drs. Ssengooba F., Kiwanuka S. and Kirunga-Tashobya C. for providing meaningful insights during the data collection and analysis; Sarah Roger, Emmy Arnold, and the various research assistants for their participation in the data collection, analysis and report writing.

## Ethical issues

This study was approved by the McMaster Research Ethics Board and Makerere University School of Public Health Research Ethics Board and the Uganda National Council for Science and Technology. All respondents provided signed consent.

## Competing interests

Author declares that she has no competing interests.

## Author’s contribution

LK is the single author of the paper.

## Endnotes


[1] In this paper we adopt Lavis et al, 2009 understanding of evidence as “concerning experience or observations (facts) that are intended for use in support of a conclusion.”

[2] In this paper priority setting is defined as: Process of ranking health interventions for resource allocation purposes. Some of the literature uses priority setting synonymously with resource allocation. It occurs at the micro-level (bedside); meso-level (regional health authorities, health institutions, district authorities); macro-level (national government, ministry of health) and the global level.


## Supplementary files


Supplementary file 1. Ministry of Health Departments and Programs.

Supplementary file 2. Interview Guide for the Retrospective Interviews With Policy-Makers (for Example HIV Theme Officer).

Supplementary file 3. In Vivo Node Tree (Expanding on the Evidence Node).

Supplementary file 4. Additional Quotes.

## Key Messages

**Implications for policy makers**

- Since it improves the quality of priority setting decisions, evidence is critical when setting healthcare priorities.
- Although there are global efforts to strengthen the evidence base for priority setting, decision-makers in low-income countries (LICs) still experience constraints in accessing and using evidence in priority setting.
- The availability, accessibility and use of evidence varies between programs depending on whether or not the program is politicized or donor supported.
- The infrastructure and resources that are available to the programs that are well-supported should be leveraged to strengthen the use of evidence in priority setting within all programs in the whole healthcare system (HCS).

**Implications for the public**

Priority setting and resource allocation are critical in low-income countries (LICs) that have limited health resources. However, priority setting can easily be influenced by several unaccepted subjective factors which can be mitigated by using evidence. Many LICs have limited access to quality evidence. We found that since healthcare systems (HCSs) in LICs still have donor supported programs, these programs tend to have more accessible, quality evidence compared to the other programs. These differences imply a possibility that priority setting in the other programs is liable to being influenced by other factors. It would be beneficial and efficient to leverage the resources that are available to the well-resourced programs and use them to improve the evidence availability, quality and access for the entire HCS. The additional challenges to the use of evidence in priority setting should also be addressed so as to ensure that the generated evidence is used to improve health sector prioritization.
