# Supplementary material for: Does the Narrative About the Use of Evidence in Priority Setting Vary Across Health Programs Within the Health Sector: A Case Study of 6 Programs in a Low-Income National Healthcare System
Source: Int J Health Policy Manag. 2020 Jan 21;9(10):448–58. doi: 10.15171/ijhpm.2019.133 (PMC7719212; doi:10.15171/ijhpm.2019.133)
Supplement: Supplementary file 1 — Ministry of Health Departments and Programs. [file ijhpm-9-448-s001.pdf]

Supplementary file 1. Ministry of Health Departments and Programs

Ministry Departments	Divisions
Finance and Administration	• Minister's Offices<br>• Senior Top Management<br><br>Three Ministers, Permanent Secretary, Director General of Health Services, Director Health Services (Planning and Development), Director health Services (Clinical and Community Services)<br><br>• Administration Division, Accounts Division, Procurement and Disposal Unit, Internal Audit, ICT Unit
Planning	• Resource Center, Human Resource Development, Policy Analysis, Planning, Budget and Finance
Quality Assurance	
Nursing	
Community Health	• **Reproductive Health, Child Health,** Environmental health, Vector Borne Disease Control, Veterinary Public Health, Health Education and Promotion, Village Health Teams (VHTs)/ Community Health Extension Workers (CHEWs), Disability & Rehabilitation Division, **Non Communicable Diseases, Control of Emergencies.**
Clinical Services	• Hepatitis B Secretariat, Integrated Curative, Health Infrastructure, Pharmacy, Mental Health, Uganda National Ambulance Services
National Disease Control	• **AIDS Control Program,** Epidemiology and Surveillance, Jiggers Eradication Program, National Onchocerciasis Control Program, Natural Chemotherapeutics Research Institute (NCRI)
