# Supplementary material for: Does the Narrative About the Use of Evidence in Priority Setting Vary Across Health Programs Within the Health Sector: A Case Study of 6 Programs in a Low-Income National Healthcare System
Source: Int J Health Policy Manag. 2020 Jan 21;9(10):448–58. doi: 10.15171/ijhpm.2019.133 (PMC7719212; doi:10.15171/ijhpm.2019.133)
Supplement: Supplementary file 3 — In Vivo Node Tree (Expanding on the Evidence Node). [file ijhpm-9-448-s003.pdf]

Supplementary file 3. In Vivo Node Tree (Expanding on the Evidence Node)

Priority setting process within low income countries

- Strategies
- PS process
- Stakeholders
- Internal influences
- Implementation
- External influences
- **Evidence**
  - **Why is it prioritised**
  - **Type**
    - **Epidemiology**
    - **Cost-effectiveness**
    - **qualitative**
  - **Role in PS**
  - **Ranking**
  - **Presented by who**
  - **How they speak of it**
  - **Sources**
  - **Evaluation and monitoring**
  - **Challenges**
    - **Availability and access**
      - **Geographical**
      - **Physical infrastructure**
      - **Political instability and natural disasters**
      - **Financial and human resources**
    - **Actual use of evidence**
      - **Poor quality/ not credible**
      - **Completeness**
      - **Timeliness, outdated**
      - **Others**
        - **Self- interest**
          - **Donor, politicians, individual**
        - **Pressure to implement**
          - **Timing**
          - **Opportunity costs**

- Equity
- Emergencies
- Crowding out
- PS Criteria
- PS Challenges
