# Supplementary material for: Does the Narrative About the Use of Evidence in Priority Setting Vary Across Health Programs Within the Health Sector: A Case Study of 6 Programs in a Low-Income National Healthcare System
Source: Int J Health Policy Manag. 2020 Jan 21;9(10):448–58. doi: 10.15171/ijhpm.2019.133 (PMC7719212; doi:10.15171/ijhpm.2019.133)
Supplement: Supplementary file 4 — Additional Quotes. [file ijhpm-9-448-s004.pdf]

Supplementary file 4. Additional Quotes

Sub-category	Illustrative Quotes
Definition of evidence	Commenting on the relevance of qualitative evidence:<br>*"...ABC is the one that caused a bit of conflict. People like it because it is a soft tactic. You didn't have to measure it. People just have meetings, I talk to them and I counsel them about abstinence, you didn't need to measure. So that one may have caused a little bit of conflict, but, on our side, it was you know we needed evidence to see if it was effective. (DAP_HIV_3)*
Sources of evidence	For example, an interviewee spoke of how national documents are used to inform her organization's priorities;<br>*"... we always depend on the documents that [the Minister of Health and National TB Program] have produced like the strategic plan and annual plan.... it's also usually good to refer to what has been documented because that way you have strong backing for while you make certain decisions." (DAP_NCD_3)*<br>*"...I think they are looking at indicators that were set either in the work plan or in the health sector strategic investment plan. So if they look at the indicators they see that for instance that maternal, child health has improved a little but they can see that maternal health is not doing well so that informs them to say you see the next item for us the priority should be maternal health." (DAP_HIV_4)*<br>Research:<br>*"Yea it is part of our main beneficiary, client beneficiary assessment. It's done through various means. Through informal, as well as well-structured (mechanisms) like suggestion boxes in the health facilities, exit interviews in some clinics but also some community level surveys to look at how programs are performing and whether people are satisfied, whether they're satisfied with us..." (MOH_HIV_2)*

	Importance of local evidence:<br>*" … I think we need to further develop a more systematic process for gathering evidence and analysing it and then bring an appraisal priority setting with input from local data" (MOH_Emergency_2).*<br>*"…First of all, for any priority setting and implementation, there should be indicators and sources of data and so there is… the self-assessment that we do here, like technical review, the annual review reports, but also there are studies, regular studies, you could get the information from DHIS, that's social information, Health Management Information System. You can definitely assess the progress of implementation of priorities…"(D3_11)*<br>*Sources: Experts*<br>*"… We still hold consultative meetings through the gathering of information that would bring stakeholders from districts, from the donor community, civil society and … pick as many topics as possible to discuss when you have the political will also there in the districts they tell you exactly what they hear from their people." (MOH_HS_1)*<br>*Additional district sources:*<br>*"…Before we set our priorities, specifically for the financial year 2015/16 we were in a position to get to learn from the District Councilors on some of the priority activities they have at their places where they represent. And this was a guiding tool on how we could craft out the work plan that can be used to be implemented in this running financial year. And I think, the Councilors being representatives of the communities where they come from, they see and hear what their residents say..." (D3_1)*
Role of evidence	*Evidence for effectiveness and advocacy*

	"…we kind of built up an evidence base where we had done work on male circumcision, where we had also tracked issues on condom use and brought that evidence to the table and presented this both to government and donors and said ok in a resource constrained environment you would be more likely to achieve results this way. And also for advocacy because it's not enough to tell the President that we want to circumcise but (we need) to take him through what about circumcision is effective..." (DAP_HIV_4
Challenges related to accessing evidence	Without adequate funding, the generation and use of evidence by sub-national and even national priority setters is less likely.<br><br>*"...For some things there is enough evidence for others, in fact for some there is too much evidence and for some the evidence to drive international policy is from here. Ok. The whole iCCM agenda is driven by Uganda. The whole HIV AIDS thing is, Uganda's been the main influence…"(MOH_MNCH_8)*<br>*"...when the Ministry of Health… I think about four years ago… wanted to conduct a non-communicable disease factor survey to establish baseline data, they contacted us at the School of Public Health to provide technical support to the Ministry to be able to conduct the survey…" (MOH_NCD_ 2)*
Challenges related to actual use of evidence	"*…So is I think the process is okay, except sometimes we also become overwhelmed in the sense that we should be looking at a thing from a technical perspective – from a perspective where equitable service are delivered based on the data you have…but we work in an environment where the political leaders are the owners of the resources… so sometimes we want to put things from a technical perspective and they want to put it from a political perspective …"(D1_5)*
